# Immune Reconstitution After Gene Therapy Approaches in Patients With X-Linked Severe Combined Immunodeficiency Disease

**DOI:** 10.3389/fimmu.2020.608653

**Published:** 2020-11-27

**Authors:** Elena Blanco, Natalia Izotova, Claire Booth, Adrian James Thrasher

**Affiliations:** ^1^Molecular and Cellular Immunology, Great Ormond Street Institute of Child Health, University College London, London, United Kingdom; ^2^Department of Paediatric Immunology, Great Ormond Street Hospital NHS Trust, London, United Kingdom

**Keywords:** X-linked severe immunodeficiency disease, gene therapy, immune reconstitution, clinical trial, T cells, B cells, NK cells, conditioning

## Abstract

X-linked severe immunodeficiency disease (SCID-X1) is an inherited, rare, and life-threating disease. The genetic origin is a defect in the interleukin 2 receptor γ chain (*IL2RG*) gene and patients are classically characterized by absence of T and NK cells, as well as presence of partially-functional B cells. Without any treatment the disease is usually lethal during the first year of life. The treatment of choice for these patients is hematopoietic stem cell transplantation, with an excellent survival rate (>90%) if an HLA-matched sibling donor is available. However, when alternative donors are used, the success and survival rates are often lower. Gene therapy has been developed as an alternative treatment initially using γ-retroviral vectors to correct the defective γ chain in the absence of pre-conditioning treatment. The results were highly promising in SCID-X1 infants, showing long-term T-cell recovery and clinical benefit, although NK and B cell recovery was less robust. However, some infants developed T-cell acute lymphoblastic leukemia after the gene therapy, due to vector-mediated insertional mutagenesis. Consequently, considerable efforts have been made to develop safer vectors. The most recent clinical trials using lentiviral vectors together with a low-dose pre-conditioning regimen have demonstrated excellent sustained T cell recovery, but also B and NK cells, in both children and adults. This review provides an overview about the different gene therapy approaches used over the last 20 years to treat SCID-X1 patients, particularly focusing on lymphoid immune reconstitution, as well as the developments that have improved the process and outcomes.

## Introduction

Severe combined immunodeficiency diseases (SCIDs) are a group of life-threatening and rare primary immunodeficiencies, with an overall estimated incidence of ≈1:50,000 live births. SCID patients are characterized by a profound defect in the number or function of T-cells, which are associated to a variable extent with abnormalities in B and NK cell populations. These immune alterations are caused by a monogenic defect, with up to eighteen different genes identified so far (1). The most common form of SCID in Western geographies is X-linked SCID (SCID-X1), which accounts for ≈25–40% of all diagnosed SCIDs (2, 3). Due to severe immune defects and the ensuing occurrence of life-threatening infections, SCID-X1 is usually lethal in the first year of life unless definitive immune reconstitution is achieved. Nowadays there are two possible therapeutic approaches for the restoration of the immune system in SCID-X1 patients: 1) hematopoietic stem cell transplantation (HSCT); and 2) autologous gene therapy (4).

Gene therapy employs the introduction of DNA or RNA into target cells by adding a correct version of the gene (additive gene therapy) or more precisely correcting the defective gene or targeting the transgene to desirable genomic sites (gene editing) (5). Gene therapy used for SCID-X1 clinical trials so far has been based on the use of retroviral vectors to transfer a corrective copy of the defective gene to autologous hematopoietic stem and progenitor cells (HSPCs), and was successfully performed for the first time more than 20 years ago (6). However, the outstanding results observed regarding the clinical benefit and the T-cell reconstitution were tempered by the occurrence of leukemogenesis in some patients (7). With the development of safer vectors as well as the introduction of low-dose conditioning regimens, new clinical trials are currently being performed to confirm the safety and effectiveness of these therapeutic approaches. Here, we review the main characteristics of SCID-X1 and the available treatments, especially focusing on the technical aspects of gene therapy approaches used over time, and the resultant immunological reconstitution of T, B, and NK cells.

## Physiopathology of SCID-X1

The genetic origin of SCID-X1 was identified in 1993 due to the presence of mutations in the interleukin 2 receptor γ chain (*IL2RG*) gene (8, 9). This gene is located on Xq13.1 locus thus has an X-linked inherited pattern and all patients are males (10). The *IL2RG* gene contains eight exons and more than 200 unique mutations have been identified so far (11). Around one half of mutations are missense and nonsense, while the remaining half are insertion/deletions or splicing mutations (11).

The *IL2RG* encodes the common cytokine receptor γ chain (γc), which is part of the receptor for a number of cytokines, including IL-2, IL-4, IL-7, IL-9, IL-15, and IL-21 (12). Due to the critical role of IL-7 and IL-15 in T-cell and NK-cell maturation, respectively (13), SCID-X1 patients are typically characterized by the absence of T cells and NK cells. In addition, despite the normal or even elevated number of B cells observed in SCID-X1 patients, these are only partially functional, most likely due to the defective signaling of IL-4 and especially IL-21 (14). Accordingly, these patients classically present with defects in both humoral and cellular compartments of the immune system, and a T-B+NK- phenotype (15). Without a curative treatment, patients usually succumb early in life to viral and opportunistic infections (4, 10). Nonetheless, some forms of atypical SCID-X1 with milder phenotypes have been identified, most of them caused by hypomorphic mutations (11, 16) and others as a result of partially corrective somatic reversions (17–21).

The early treatment of patients, achieved through earlier diagnosis, is associated with a better outcome (2). Thus, neonatal screening for SCID based on the T cell receptor excision circle (TREC) assay is being applied in many countries worldwide either as pilot studies or incorporated into routine healthcare (2, 22). The identification of reduced or absent TRECs can also be caused by non-SCID diseases (2), so this finding must be followed by lymphocyte immunophenotyping and further diagnostic investigations (23, 24) to help orientate the genetic studies (15). Due to the presence of maternal T-cells or leaky production of oligoclonal cells, total T-cell numbers might initially be significant, so the analysis of subpopulations including naïve T-cells and recent thymic emigrants (RTE) is crucial (23, 25). The final diagnosis of SCID-X1 is established by the identification of pathogenic variants in the *IL2RG* gene, although sometimes this requires confirmation by other studies, such as functional assays, especially in atypical SCID-X1 (26). The expression of γc is not conclusive, as it can be normal (but nonfunctional) in some patients (10).

## Treatment Approaches

Following a diagnosis of SCID-X1, therapeutic measures must be applied as soon as possible, including transfer to a specialized center, establishment of immunoglobulin replacement therapy (IgRT) and appropriate antimicrobial prophylaxis (15, 27–30). HSCT or gene therapy should be performed as soon as possible to restore immunity, for instance adhering to the consensus guidelines proposed by the European Society for Blood and Marrow Transplantation and the European Society for Immunodeficiencies (EBMT/ESID) (31) or USIDnet advice.

### Hematopoietic Stem Cell Transplantation

Since the first SCID-X1 patient was successfully treated with HSCT in 1968 (32), this approach has been the treatment of choice for many forms of PID (33). Despite a relatively high number of reports showing the results obtained after HSCT in SCID patients, and differences in the survival and immune recovery according to the SCID subtype (34, 35), very few studies focused specifically on SCID-X1 (36, 37). Overall survival of SCID patients after HSCT is >70% (34, 35, 38), although several factors may have an impact, such as donor matching, older age, presence of infection, SCID phenotype/genotype and ethnicity (34, 35, 38, 39). Use of geno-identical matched sibling donors (MSDs) results in the highest survival rates (>90%) (34, 35, 38, 40, 41). However, because MSDs are available for less than 20% of SCID patients, alternative donors including mismatched related donors, matched unrelated donors or umbilical-cord blood donors are often used, with lower overall survival rates (≈60-75%) (34, 35, 38, 41). Overall survival rates using these alternative donors have however increased considerably over the years, most likely due to the improvement in HLA-typing techniques as well as the use of treatments to abrogate complications such as graft versus host disease (GvHD) (42, 43). Accordingly, T-cell and B-cell reconstitution is usually superior in patients treated with MSDs *vs.* other donors (38). Independent of the type of donor used, HSCT performed in patients with age <3.5 months is associated with a higher survival and reduced rate of clinical problems (38, 39). On the other hand, the presence of active infection is associated with reduced survival (38). Other complications that affect post-HSCT outcomes include acute and chronic GvHD, graft failure requiring a second transplant, and late effects of conditioning regimens (34, 38).

Immune reconstitution after HSCT is usually achieved in the T cell compartment after 3–4 months, normalizing after 9–12 months (43). The numbers of CD4^+^ and CD4^+^ CD45RA^+^ naïve T cells early after HSCT are predictive of long-term reconstitution and overall survival (34, 44). In contrast, B-cell immune recovery is more variable, with 43%–66% of SCID-X1 being dependent on IgRT (36, 39, 41, 45), which has been recently associated with a poorer quality of life in these patients (36). The lack of B-cell functional recovery observed in SCID-X1 patients despite successful T-cell reconstitution is likely due to a failure in the signaling through IL-21 in B-cells (14) and is associated with a reduced donor B-cell engraftment particularly in unconditioned procedures (38, 45–48). Nevertheless, a recent study reported an ongoing B-cell response with intact host B-cell signaling through IL-21 in a SCID-X1 patient 47 years after the HSCT, although this is most likely due to a hypomorphic γc variant (49).

Reduced B-cell engraftment and reconstitution in SCID-X1 patients might be explained by the occupation of niches by host B-cell precursors, as in these patients the B-cell maturation in bone marrow (BM) is unaffected (50). Therefore, the use of pre-transplantation conditioning (typically alkylating agents such as busulfan) has been applied in several centers (46). On the one hand, it is clear that pre-conditioning treatment is associated with a lower risk of treatment failure and more reliable B-cell reconstitution, which is associated with a better outcome (34, 38). However, some authors claim that this approach is not a guarantee for recovery of B-cell function, since there are cases without conditioning that maintain B-cell chimerism, as well as patients with pre-conditioning that still need IgRT after HSCT (46, 51). In addition, some studies suggested a relationship between the use of conditioning and reduced survival (51), even though this has not been universally observed in other multicenter studies (34, 35), as well as a potential risk of GvHD (34). Similarly to B cell recovery, successful NK cell reconstitution has been associated with pre-conditioning treatment (52).

In conclusion, pre-conditioning seems to improve B-cell chimerism and immune reconstitution in SCID-X1 patients after HSCT (34, 36, 37, 53), although more studies are needed to determine the ideal regimen balancing efficacy with toxicity, particularly in young infants (54). In line with this, the possible use of targeted biologic agents for stem cell depletion (e.g. monoclonal antibodies such as anti-CD117 or anti-CD45) is very promising (55–57).

### Gene Therapy

**Figure 1:**
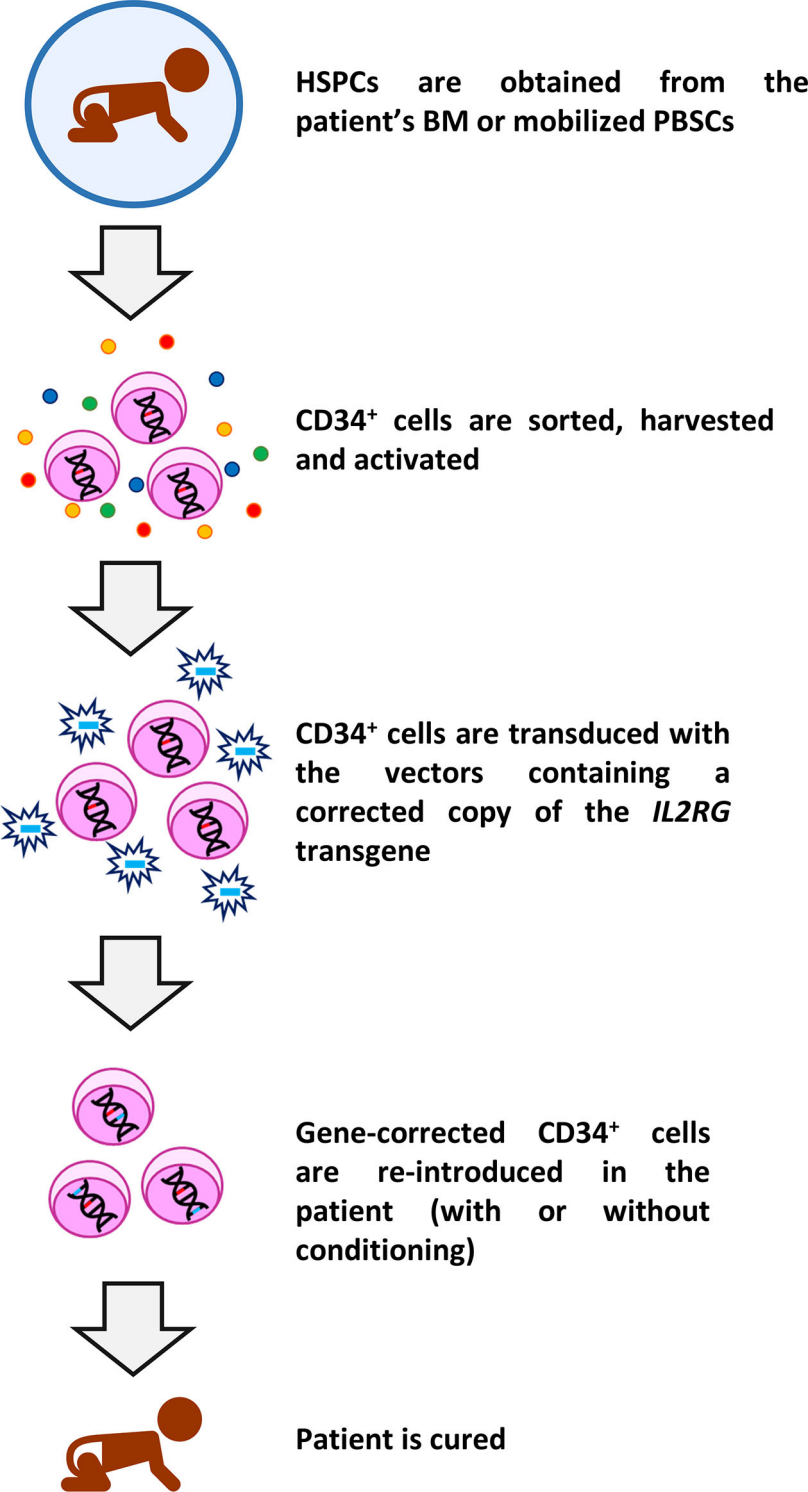
Schematic representation of gene therapy protocol used in SCID-X1 clinical trials. CD34^+^ HSPCs are sorted from bone marrow (BM) or mobilized peripheral blood stem cells (PBSCs) using magnetic separation and cultured with cytokines. Thereafter, cells are transduced with the specific vector containing the corrective human interleukin-2 receptor γ gene (*IL2RG*) and after a period of culture, the cells are re-infused in the patients. In most recent clinical trials, the cells are infused after a low-dose conditioning treatment. Subsequently, the immune reconstitution starts, and the patient is theoretically cured for life.

Despite the undoubted benefit of HSCT in the treatment of SCID-X1, the difficulties to find a suitable donor, lower survival rates in the absence of MSD, lifelong use of IgRT in a relatively high number of cases, as well as the occurrence of adverse effects like GvHD and graft failure/rejection have driven the development of autologous gene therapy as an alternative curative treatment. In SCID-X1 patients, all the procedures clinically used so far consist of *ex vivo* introduction of a corrective copy of the *IL2RG* gene using viral vectors in autologous HSPCs (6, 58–64). In brief, CD34^+^ HSPCs are obtained from BM or mobilized peripheral blood stem cells and cultured and transduced under conditions that enhance permissiveness to gene transfer. Finally, the corrected cells are re-introduced in the patients, either with (in latter studies) or without any kind of prior conditioning. A schematic representation of gene therapy protocol used in SCID-X1 patients is shown in **Figure 1**.

Gene therapy is particularly attractive in the case of SCID-X1, due to the powerful survival and growth advantage of γc corrected precursors (65). This is in line with the partial but sustained immune reconstitution of T-cells observed in SCID-X1 patients with spontaneous revertant mutations in their precursor cells (18–21, 66, 67). In general, these reversions are limited to the T-cell compartment (18, 19, 21), despite some exceptions that have been recently described (20, 66, 67). Hsu et al. reported a patient with a reversion observed in T cells and also in B cells, although only ≈1% of B cells expressed γc and this was not enough to correct the antibody deficiency (20). This reduced percentage might be the result of the lack of (or reduced) advantage for corrected B-cell precursors due to the redundant role of γc in antigen-independent B-cell maturation, though this is indispensable for its function (14, 68). Furthermore, Okuno et al. reported another patient with a probable reversion, detected in several lymphoid subsets, showing the highest percentages of reversion in CD8^+^ T-cells (19%) and NK cells (31%). Thus, authors suggest that the correction might have occurred in a common T/NK precursor and these cytotoxic cells would have a growth advantage (66).

**Figure 2:**
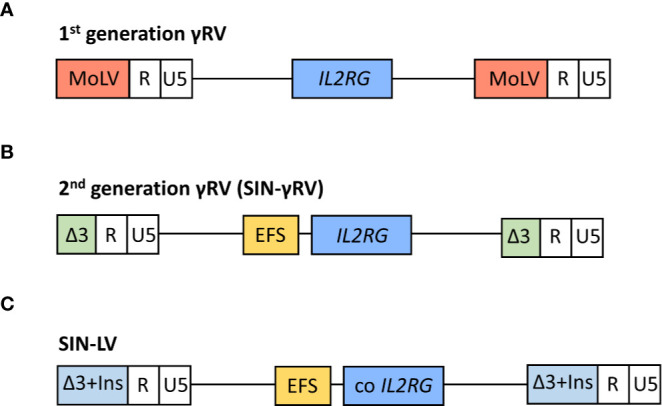
Schematic representation of viral vectors used in the different clinical trials for the treatment of SCID-X1 patients. In 1^st^ generation γ retroviral vectors (γRVs), the gene expression is controlled by viral long terminal repeat (LTR) sequences **(A)**. In 2^nd^ generation self-inactivating (SIN) γRV and lentiviral vectors (LVs), the U3 region of LTR is deleted and the human interleukin-2 receptor γ gene (*IL2RG*) expression is driven by an internal mammalian promotor **(B)**. Moreover, the LVs contain a codon optimized (co) *IL2RG* complementary DNA to further improve the transgene expression, and U3 region is replaced with a chromatin insulator element (Ins) **(C)**. MoLV, Moloney murine leukemia virus; EFS, eukaryotic human elongation factor 1α (EF1α) short promoter.

Gene therapy was first implemented in SCID-X1 patients because of the encouraging results obtained in *in vitro* studies (69–71) and animal models (72) by *IL2RG* gene transfer using gammaretroviral vectors (γRV). The first gene therapy clinical trial in patients with SCID-X1 began at Necker Hospital (Paris, France) in 1999 (6, 58), followed soon after by another clinical trial carried out at Great Ormond Street Hospital (GOSH, London, UK) (59). The inclusion criteria for these trials included confirmed mutation in *IL2RG* gene and the absence of an HLA-identical donor (58, 59). Patients age ranged between 1 and 46 months (73, 74). Both studies used a first-generation Moloney murine leukemia virus vector expressing the γc complementary DNA, and the transgene expression was regulated by endogenous viral long terminal repeat (LTR) sequences. A schematic representation of vectors is shown in **Figure 2A**. γRVs were chosen because these were the first retroviruses applied to hematopoietic cell gene transfer, and a random integration pattern was expected (75). Both studies followed the same protocol, with some differences in vector pseudotyping and culture conditions (59). Corrected cells were introduced in the patients without any kind of previous conditioning (6, 58, 59).

The results were exceptional, with most patients showing good long-term immune reconstitution (discussed below) and an overall survival of 90% (18/20) (taking both clinical trials together) (73, 74). However, unexpectedly six patients developed T-cell acute lymphoblastic leukemia (T-ALL) 2–14 years after the treatment (five in Paris and one in London), being fatal for one patient (7, 76–78). All the other patients responded well to the chemotherapy treatment. Interestingly, T-cell reconstitution substantially recovered after chemotherapy without the immediate need for a further autologous or allogeneic graft (73, 74). The origin of this effect was insertional mutagenesis, due to the integration of the vector near proto-oncogenes (mainly *LMO-2*) and their transcriptional activation by the viral LTR enhancer sequences (76, 77, 79). In addition, the presence of other multiple genetic aberrations suggests that the insertional mutagenesis was the first event and was followed by other hits that contributed to the malignant transformation (76, 77). At first, aberrant γc signaling was thought to contribute to leukemogenesis (79), which would be in line with the absence of clonal proliferation in adenosine deaminase (ADA) deficiency SCID (ADA-SCID) patients (80, 81), despite similar integration pattern near *LMO2* and other proto-oncogenes in these patients (82). However, neither γc overexpression nor constitutive activation of JAK3 was observed in the leukemic cells of SCID-X1 patients (76, 79). Despite these results, the correction of *IL2RG* was suggested as a contributor itself to cancer development, based on murine models (83, 84). However, this hypothesis is controversial and several papers have strongly argued against this concept (85–87) [Pike-Overzet et al. (65) reviewed exhaustively the main findings regarding this issue], and it is now commonly accepted that *IL2RG* does not have an inherent oncogenic role although it is clearly permissive to T cell development. Accordingly, a recent study using transgenic mice showed that *IL2RG* was not directly oncogenic, although an accelerated leukemogenesis was observed in the thymus due to the functional cooperation between *LMO2* and *IL2RG* (88), indicating that *IL2RG* might have a cooperative role. Self-renewal of thymocytes induced by the limiting thymic precursor supply from BM has been also suggested as another contributor to T-ALL in these patients (89, 90), as in these subjects T-cell production is maintained in the absence of corrected precursors in BM (73). However, these studies are based on murine models and do not appear to be supported by clinical experience in unconditioned allogeneic HSCT where leukemogenesis has not been observed (91). In conclusion, the leukemogenesis observed in some SCID-X1 patients treated with gene therapy predominantly originates from insertional mutagenesis associated with the γRVs, which (contrary to the previous belief) do not have a random integration pattern (92). Detailed insertion site analysis has demonstrated a preferential integration in transcriptionally active gene regulatory regions. This favored target site profile has its origin in the interaction between the virus integrase and the bromodomain and extraterminal domain proteins (BET), which also interact with histone acetyl modifications in active enhancers and promotors (93–95). Therefore, these vectors preferentially target active genes, and can cause gene expression dysregulation induced by endogenous proviral promotor and enhancer sequences located in the proviral LTRs. Subsequently, leukemogenesis has also been observed in patients with Wiskott-Aldrich syndrome (96) and chronic granulomatous disease (97) treated with similar vector configurations. In contrast, despite the use of γRVs for gene therapy of ADA-SCID and the similar vector integration profile observed (98), no malignant transformation has been reported in any of these patients so far (80, 82, 99, 100). A possible explanation for this phenomenon is that a more detoxified environment, as a consequence of the recovery of ADA function, would facilitate the maturation of non-corrected thymocytes, which might compete with transduced cells containing the deleterious mutations (90). On the other hand, the improved engraftment achieved by conditioning treatment used in ADA-SCID patients could lead to increased thymus precursor seeding from bone marrow HSPC, reducing the thymocyte replicative stress and consequently the oncogenic risk (90). Conditioning may, therefore, play an important role, not only in the efficacy of the therapy (discussed below) but also in safety.

Consequently, extensive efforts were made to develop safer vectors, reducing the insertional activation of endogenous genes but maintaining efficacy. The first approach was the design and evaluation of self-inactivating (SIN)-γRVs, in which the LTR U3 regions (containing the viral promoter and enhancer sequences) were deleted, and transgene expression was controlled by internal regulatory elements (101, 102). Thus, a SIN-γRV with *IL2RG* expression driven by the eukaryotic human elongation factor 1α (EF1α) short promoter (EFS) (**Figure 2B**) was used in a multicenter clinical study performed in Europe and USA (63). Enrolled patients were infants with SCID-X1 and inclusion criteria included confirmed mutation in *IL2RG* gene and the absence of an HLA-identical donor or the presence of therapy-resistant infection (this last criterion was mandatory for patients from France) (63). As well as in previous SCID-X1 clinical trials, corrected cells were infused in the absence of conditioning (63) (except for the final unreported patient treated who received low dose busulfan) (103). Data published in a preliminary report in 2014 (63) included nine patients <1 year old and the results demonstrated a similar immune recovery compared with the first clinical trials using 1^st^ generation γRVs and an encouraging survival rate of 89% (8/9). Importantly, the integration profile analysis showed a lower frequency of integration sites near cancer-associated genes in this second clinical trial *vs.* previous studies. As expected, similar integration patterns were observed for 1^st^ generation γRVs and SIN-γRVs (63, 104), in line with the described preferential integration patterns mediated by the viral integrase (93–95). Differences in overall profile therefore most likely result from *in vivo* selection of clones containing insertions near growth-promoting genes mediated by the enhancer sequences located in the LTR (63). Accordingly, after a median follow-up of 7.9 years (2.7–9.3 years), none of the SCID-X1 patients treated with SIN-γRVs have to date developed cancer (103), so the improved safety of these vectors observed in pre-clinical studies (101, 102) is encouraging.

More recently, SIN-lentiviral vectors (SIN-LVs) have been developed, in which codon-optimized γc expression is driven by an internal EFS promoter element (105, 106) (**Figure 2C**), together with the use of low level conditioning (60, 62). LVs have advantages because they exhibit a safer integration pattern (107) and more efficient transduction of quiescent HSPCs (108). Furthermore, SIN-LVs have shown promising results regarding efficacy and safety in the treatment of other diseases (109, 110), as well as in SCID-X1 pre-clinical studies (106, 111, 112). Similar to SIN- γRVs, the absence of viral promoters/enhancers likely play a major role in the increased safety. The use of low-dose pre-conditioning to improve engraftment and immune recovery in SCID-X1 patients was encouraged by previous gene therapy clinical trials (80, 113). Currently, several clinical trials using SIN-LVs together with the use of busulfan as a non-myeloablative conditioning are being conducted (**Table 1**) and the preliminary results reported in both children and adults have showed very promising results with respect to immune reconstitution (see below) and safety (60, 62), even though longer follow-up is needed. The absence of leukemia development so far in those patients treated with SIN-γRVs and SIN-LVs also supports the idea of *IL2RG* correction did not have an oncogenic role in SCID-X1 patients treated in the first clinical trials.

**Table 1:** Gene therapy clinical trials recruiting patients for the treatment of SCID-X1 registered in https://clinicaltrials.gov/.

Number	Estimated number of patients	Locations	Start date	Treatment	Age	Refs
NCT01306019	30	Bethesda	2012	Vector: Lentivirus Conditioning: Busulfan Other drugs: Palifermin (to prevent side effects of busulfan)	2–40 y	(62)
NCT03315078	13	Bethesda	2012	Vector: Lentivirus Conditioning: Busulfan Other drugs: Palifermin (to prevent side effects of busulfan)	2–40 y	
NCT01512888	28	San Francisco Memphis, Seattle	2016	Vector: Lentivirus Conditioning: Busulfan	<24 m	(60)
NCT03217617	10	Beijing Shenzhen	2017	Vector: Lentivirus	1 m–10 y	
NCT03601286	5	London	2018	Vector: Lentivirus Conditioning: Busulfan	2 m–5 y	
NCT03311503	10	Los Angeles Boston London	2018	Vector: Lentivirus Conditioning: Busulfan	<5 y	
NCT04286815	10	Chongqing	2020*	Vector: Lentivirus	<18 y	

*Estimated start date. m, months; y, years.

## Immune Reconstitution in SCID-X1 Patients After Gene Therapy Treatment

In general, immune reconstitution achieved in SCID-X1 patients treated with gene therapy has been encouraging, although some variability has been observed among the different clinical trials performed so far, as well as among the patients included. In the next sections we discuss the main results obtained regarding the immune system recovery after gene therapy and the possible factors affecting, as well as the differences observed *vs.* HSCT.

### T Cells

In SCID-X1 children treated with gene therapy, T-cell recovery has been observed in the majority, independent of the vector employed (6, 58, 63, 73, 74). Thus, in those patients treated with 1^st^ generation γRVs, T-cell recovery was observed for 19/20 patients around 2–5 months, with 13/20 achieving levels of T-cells comparable to age-matched healthy donors at 6 months (58, 59). However, two patients received a subsequent HCST at 7 and 26 months after gene therapy, due to sub-optimal immune reconstitution and clinical deterioration (58, 114). One of these patients (114) as well as one of those that developed T-ALL died (74). The remaining 17 patients continue to be followed-up: 15/17 patients showed normal or near normal T-cell numbers, including CD4^+^ T-cells (10/17) and CD8^+^ T-cells (16/17), according to the last published report (GOSH cohort: 4.5–8.9 years; Necker cohort: 7.3–10.7 years) (73, 74). Strikingly, most patients from both studies showed a reduced CD4:CD8 ratio (ranging from 0.19 to 1.0) at the last follow-up (73, 74), which has been also reported in patients with *IL2RG* spontaneous reverse mutations (18–21). It has been hypothesized that this might be result of a skewed selection of CD8 cells in the thymus or a consequence of antigen-driven response, although the reason of this phenomenon remains elusive.

Similar results were obtained for the patients treated with 2^nd^ generation γRVs, without significant differences *vs.* previous clinical trials (63). From nine patients included in this study, seven were followed-up over 1–3.2 years (one patient died because of a pre-existing infection and the other was removed from the trial and underwent a cord blood transplant, due to the absence of marked T-cells). From these seven infants, six achieved a sustained CD3^+^, CD4^+^, and CD8^+^ T cell recovery. Regarding the SCID-X1 infants treated with LVs and low-intensity conditioning treatment, preliminary reports have shown very promising results, with rapid recovery of T-cells (including CD4^+^ and CD8^+^), reaching stable and normal values around 6 months in 7/8 cases (60).

Interestingly, the presence of naïve T-cells in SCID-X1 patients years after the gene therapy (with normal or slightly reduced percentages in most of the cases), which correlated with the number of TRECS (63, 73, 74), suggest ongoing T-cell production in these patients. These naïve T-cells were detected despite the lack of transduced B and myeloid cell, which indicates the absence of transduced common progenitors in BM and a persistence of long-term T-cell progenitors instead (74), in line with the results recently reported by Izotova et al. (discussed below) (115).

Polyclonality was demonstrated in most children treated, independently of the vector employed, in both the TCRvβ family distribution and the CDR3 lengths (60, 63, 73, 74). Some patients showed a more restricted repertoire, which mirrors suboptimal immune reconstitution, especially in terms of total T-cells and naïve T-cells (60, 73). With regard to the functional capacity of T-cells, proliferative response to PHA, anti-CD3 or specific antigens (after immunization or infection) was recovered and maintained over time in most patients, although subnormal values were observed in some (63, 73, 74).

**Figure 3:**
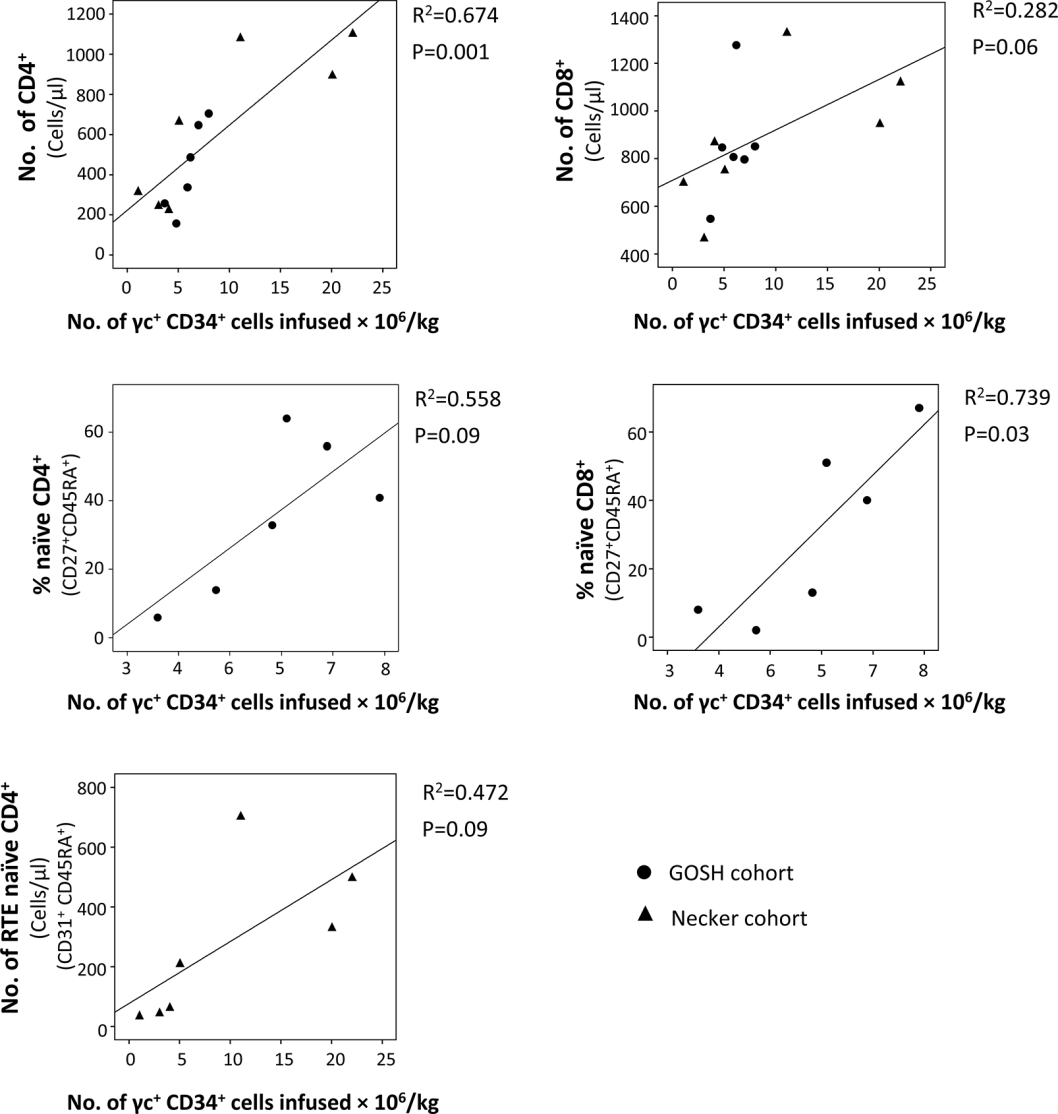
Correlation between the number of γc^+^ CD34^+^ cells infused and T-cell subsets in the last follow-up reported. Data were obtained from patients treated with the 1^st^ generation γ retroviral vectors (γRVs) (73, 74). When it was possible (same variables were reported or it was possible to calculate them with the information provided in the original publications), both cohorts (Necker and GOSH) were analyzed together. Correlations were determined by (squared) Pearson correlation coefficient using SPSS v18 software (IBM SPSS Statistics, IBM, Armonk, NY).

Despite the overall good T-cell immune reconstitution achieved in SCID-X1 children treated with gene therapy, some variability between patients has been observed. Numbers of γc^+^ CD34^+^ cells infused seems to be an important factor that affects the immune recovery (75), since some of the patients treated with 1^st^ generation γRVs who received lower dose of corrected cells had the poorest T-cell recovery with two of them needed subsequent HSCT (73, 74, 114). In fact, taking the data from these two cohorts (73, 74), significant correlations between the number of CD34^+^ γc^+^ cells infused/Kg and the percentage of naïve T-cells CD8^+^ (P=0.03) and the number of T-cells CD4^+^ (P=0.001) were observed at the last follow-up (**Figure 3**). Cavazzana et al. established an approximated minimum of 3x10^6^ CD34^+^ γc^+^ cells/Kg to achieve successful immune reconstitution (116). Vector copy number (VCN) in the graft was also been demonstrated to be important. In the cohort of patients treated with 2^nd^ generation γRVs vectors, patients with a VCN >0.7 in the graft showed more reliable cell recovery (63). CD34 expression in the graft might also play a role in T-cell reconstitution. In a recent report of SCID-X1 infants treated using LVs, two patients showed reduced expression (mean fluorescence intensity) of CD34^+^ in the graft prior to transduction, which was related to relatively low yields in both cases (reduced VCN and CD34^+^ doses). However, only one of them showed poor T-cell recovery (60).

Clinical complications at the time of gene therapy, such as hemorrhagic events (73) or viral infections (114), have been also suggested to have a role in the immune recovery. However, normal reconstitution has been observed in other SCID-X1 cases with infections at the time of gene therapy (114). The presence of active infection at the time of HSCT in SCID patients has been also related to poor T-cell recovery in some studies (38), but not in others (34). On the other hand, in one of the treated infants in whom gene therapy failed, authors suggested that an atypical T-B+NK+ phenotype could have hampered the selective growth advantage of corrected T-cell progenitors in BM, hindering the T-cell recovery (114). Interestingly, a NK+ SCID phenotype has been suggested to be related with a poorer long-term T-cell recovery after HSCT *vs.* NK- (and NK^low^) phenotypes (38, 117). However, the presence of NK cells before the gene therapy did not seem to be an impediment for the T-cell recovery in other cases (60, 63), indicating the need for more systematic evaluation of this phenomenon.

Older age at the time of gene therapy is clearly associated with diminished capacity for T-cell recovery. Among SCID-X1 children treated with gene therapy, modest T-cell reconstitution was observed in the oldest patient (3.8 years old) (59, 73), in line with results observed in other gene therapy clinical trials performed in adolescents and adults (61, 62, 64). Thus, the results obtained in five patients with attenuated forms of SCID-X1 or previous long-term failed HSCT, and ages ranging from 10 to 20 years old treated with BM CD34^+^ or GCSF-mobilized PB CD34^+^ transduced with 1^st^ generation γRVs in the absence of conditioning, were relatively poor (61, 64). Limited if any T-cell reconstitution was observed in 4/5 cases (without naïve T-cells and extremely low values of TRECS) (61, 64). In the other case (10 years old at the time of gene therapy), a discrete increase of T-cells was observed, with presence of TRECs and recovery of proliferation capacity, but without reaching normal values for any T-cell subset (61). Strikingly, this case had showed a transient T-cell recovery after BM transplantation. Consequently, authors suggest not only an age-dependent effect, but also previous (thought temporal) thymopoiesis as an important factor for the success of gene therapy in these older patients (61). More recently, results from a clinical trial performed in five SCID-X1 patients treated with LVs and low-dose of conditioning aged between 10 and 23 years old were reported. Interestingly, those patients followed over time showed an increase in the number of marked T-cells and TRECS (62), so the combination of LVs and reduced-intensity conditioning treatment seems to achieve some T-cell reconstitution even in older patients. However, T-cell recovery was slower *vs.* infants treated with a similar approach (60), emphasizing the importance of more preserved thymic function.

Finally, recent reports tracking T-cells through the evaluation of vector insertional sites and TCR rearrangements in SCID-X1 patients after gene therapy has provided valuable information about immune reconstitution from gene-modified progenitors (115, 118). Clarke et al. estimated a minimum population of 144-6,018 active progenitors in patients treated with γRVs (follow-up: 4–180 months), with those cases who had suffered T-ALL showing smaller population sizes. Authors suggest that the occurrence of T-ALL and chemotherapy treatment might have diminished the pool size (118), which is in line with the lower number of naïve T-cells detected in one SCID-X1 patient treated with gene therapy that had suffered T-ALL (115). T-cell diversity, usage of V and J gene segments in TCR-beta and the number of unique TCR-beta CDR3 sequences were similar in healthy donors and almost all patients, despite authors suggested that a slow but gradual loss of diversity might be occurring in those patients with longer follow-up (118). Furthermore, the minimum number of divisions to generate the observed TCR-beta cell population from the precursors was estimated ≈9 (118). Most recently, Izotova et al. analyzed the clonal diversity and insertional site sharing of T-cell subpopulations over the time in SCID-X1 patients treated with 1^st^ generation γRVs. *De novo* production of true (functionally and phenotypically) naïve T cells from long-term lymphoid progenitors (LtLP) was demonstrated up to 15 years after the loss of gene-modified HSPCs, as well as an ongoing differentiation of naïve T cells into memory/effector T-cells. T-cell production was estimated to be sustained by 2,092–6,056 individual engineered LtLP clones, as previously predicted by Scala et al. (115, 119).

### B Cells

In contrast to the outstanding T-cell recovery achieved across gene therapy clinical trials performed in SCID-X1 patients, B-cell reconstitution has been, in general, much more variable. Thus, in those SCID-X1 children treated with γRVs (1^st^ and 2^nd^ generation) in the absence of conditioning, only a partial and non-sustained recovery of B-cell function was achieved, which was in line with the absence or extremely reduced numbers of transgene marked B-cells (58, 59, 63, 73). Reduced levels of IgM and IgA were reported in ≈15%–70% and ≈30%–60% of patients, respectively, IgG evaluation being difficult to interpret because of IgRT, which is usually administrated to these patients. In addition, successful IgRT withdrawal is typically considered in itself as a marker of B-cell function correction, and 7/17 patients treated with 1^st^ generation γRVs and all treated with 2^nd^ generation γRVs remained on IgRT in the last reported follow-up (63, 73, 74). It is noteworthy that the parameters used to inform discontinuation of IgRT were not always the same, and included the T-cell numbers (74), IgA and IgM levels (59, 73), or clinical parameters (74).

Despite the widely used and undeniable utility of serum antibodies to evaluate the B-cell response, these might not reflect accurately the ongoing B-cell response (120), as antibodies are principally produced by long-living plasma cells, which are located mainly in BM niches for years or even decades (121–123). In which case, the detection of serum Ig levels over time observed in some SCID-X1 infants after gene therapy may reflect an early but temporal recovery of B-cell function. This would be in line with the initial response to vaccination observed in some patients, but generally suboptimal and non-sustained over time, and most likely linked to the decrease in the percentage of transduced B-cells (from 1% during the first 2 years to <0.1% 6–10 years after therapy) (74). Accordingly, normal serum IgM levels is not a good predictor of B-cell response in SCID-X1/JAK3 SCID patients after HSCT, despite it being a widely parameter used to decide the withdraw of IgRT (37). Consequently, alternative parameters should be also considered to evaluate recovery of B-cell function. Vaccination response is a more reliable and predictive indicator, and has been extensively measured in SCID-X1 patients after treatment, using a wide number of polysaccharide and protein antigens (37, 73, 74). However, despite its utility (124), this approach is impeded by the use of IgRT and cannot be performed in all patients. One possible solution might be the immunization with neoantigens, as the T-cell dependent ΦX174 bacteriophage, which has already been used in SCID-X1 treated with HSCT (45). On the other hand, other measures such as presence of class switched memory B-cells or *in vitro* stimulation with IL-21, have demonstrated to be very useful for the evaluation of B-cell function recovery, showing a good correlation with vaccination response (37). This might help to inform clinical decisions, such as the initiation of immunization as well as the withdrawal of IgRT (14, 125). In line with the disappointing vaccination results obtained in SCID-X1 children treated with 1^st^ and 2^nd^ generation γRVs without conditioning (63, 73, 74), memory B-cells were reduced in the last follow-up in virtually all the patients. Accordingly, no response to IL-21 was observed, despite some proliferation was detected after stimulation with CD40L and IL-4 (74), probably through the activation of IL-13 receptor (without γc) (45, 126).

**Figure 4:**
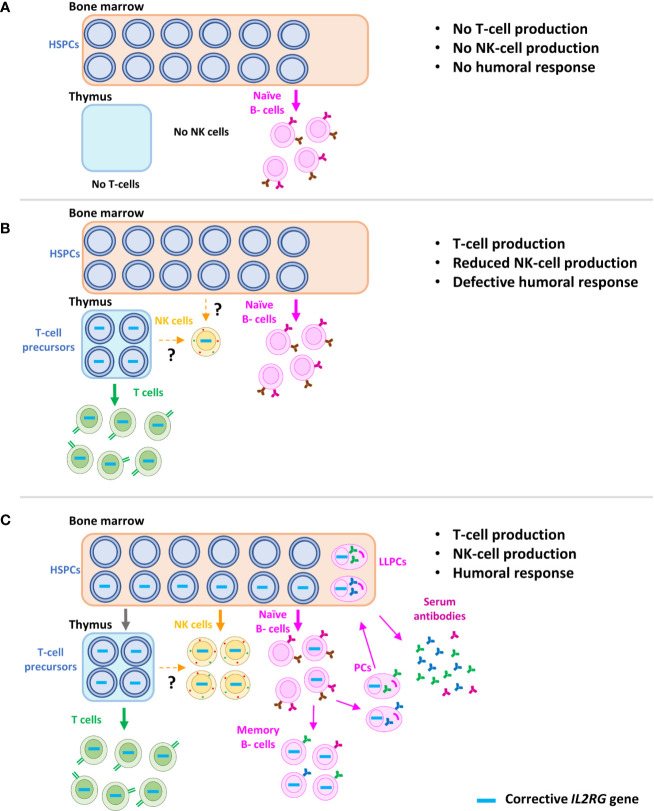
Schematic representation of immune reconstitution in SCID-X1 patients after gene therapy. Characteristic phenotype of SCID-X1 patients, with absence of T and NK cells and only presence of naïve B-cells, but without differentiation capacity (T-B+NK- phenotype) **(A)**. Immune reconstitution accomplished after the gene therapy using γRVs in the absence of conditioning treatment. Corrected progenitors seed the thymus and a sufficient reconstitution of T-cells is achieved. However, due to the loss of corrected hematopoietic stem cells in bone marrow, only a partial and non-sustained recovery of NK cells and B cells is observed. Interestingly, few marked NK cells are detected years after the therapy, most likely produced from a common long-term lymphoid progenitor, which might be in thymus or bone marrow **(B)**. Immune recovery of SCID-X1 patients after gene therapy using LVs and conditioning treatment, which favor the engraftment of corrected hematopoietic stem cells **(C)**. HSPCs, hematopoietic stem and progenitor cells; PCs, plasma cells; LLPCs, long-living plasma cells.

In summary, gene therapy based on γRVs in the absence of conditioning failed to deliver a sustained and optimal B-cell recovery, despite successful T-cell reconstitution and therefore restoration of T cell help (**Figures 4A, B**). The reason for these results is most likely the absence (or extremely low) percentages of corrected B-cell years after the treatment, due to the reduced engraftment of corrected progenitors (**Figure 4B**), and hence a maintained defect in IL-21 signaling in B-cells (14, 74, 125). This effect is also observed in those SCID-X1 patients with reduced B-cell chimerism after HSCT (37). Because of the problems linked to a defective B-cell recovery, such as higher risk of pulmonary infections and chronic lung disease, as well as the reduced quality of life associated with the dependence to IgRT (36), improvement in the B-cell reconstitution is an important consideration in SCID-X1 treatments. Therefore, according to the good results obtained using reduced-intensity conditioning in gene therapy treatment for ADA-SCID patients (81, 113), as well as in pre-clinical models of SCID-X1 (112), low-dose conditioning regimens have now been applied to gene therapy clinical trials for SCID-X1 (**Table 1**) (**Figure 4C**).

Preliminary results of trials using LVs and conditioning in SCID-X1 patients show promising results regarding B-cell function recovery. In the first clinical trial performed in five patients between 7 and 22 years old in which previous HSCT failed, marked B-cells appeared early after the infusion of corrected cells, and B-cell recovery was demonstrated by the increase of IgM from 3 months after therapy (62). Moreover, in two patients followed-up for more time (24–36 months), reconstitution of B-cell function was confirmed by the vaccination response, *in vitro* stimulation by IL-21, normal IgG after IgRT withdrawal and the presence of IgG^+^ memory B-cells. Interestingly, the humoral reconstitution corresponded with the eradication of chronic norovirus infection (62). Regarding the recovery of B-cell function in SCID-X1 infants treated with this approach, preliminary results reported by Mamcarz et al. show a normalization of IgM in 7/8 patients 3–6 months after the gene therapy, protective vaccination response in most cases evaluated and independence of IgRT in 4/8 cases (60). Vector-marked B-cells were detected at 2–3 months and VCN in B-cells remained relatively high and stable (as well as in other lineages) throughout the follow-up time (up to 18 months) (60). Interestingly, as de Ravin et al. pointed out, LVs and their capacity to target HSPCs more efficiently also might have an important role to improve the engraftment in these patients (62). In conclusion, preliminary reports of the use of LVs and low-dose conditioning treatment show very promising results with regard to B-cell reconstitution, although longer follow-up will be necessary to confirm these findings.

### NK Cells

Despite the early increase observed in NK cells 2–4 weeks after γRV-based gene therapy in absence of conditioning treatment (58, 59), this was not sustained. Thus, absolute numbers were below normal levels compared to age-matched healthy donors at last follow-up, and levels of marked NK cells also decreased over time (63, 73, 74). There is scarce information available about the phenotype or functional capacities of these corrected NK cells. Although efficient capacity to kill target cells (K562) (6, 58), other studies have reported a defective expression of NK-cell receptors such as NKp46 and NKG2D (127). Strikingly, a transient increase was also observed in patients who developed T-ALL, most likely as a secondary response to the clonal proliferation (74). Recently, Izotova et al. demonstrated the presence of a reduced number of NK cells up to 19 years after the treatment, and 15 years after the loss of corrected HSPCs (115). These NK cells showed a normal expression of several markers (Granzyme B, perforin, CD94), but a decreased percentage of CD56^dim^ CD16^+^ (which is the major subset in healthy donors), in line with previous results (127). Interestingly, due to the high levels of shared integration sites with naïve T-cells, an intriguing possibility is that these NK cells are most produced by a long-lived common LtLP (**Figure 4B**) (115). However, γRV gene therapy in the absence of conditioning did not favor the development and survival of NK cells as efficiently as T-cells (74).

As for B-cells, the introduction of pre-conditioning might improve NK cell reconstitution (73) due to the increase in numbers of engrafted HSPCs (**Figure 4C**). Accordingly, preliminary reports from trials using LVs and low-dose conditioning showed a relatively high and stable VCN in NK cells over time, showing age-matched comparable values in most cases (60, 62) and normal expression of natural cytotoxic receptors (62). Interestingly, in one patient with HPV related severe warts, a clear improvement was coincident with an increase in NK cells (62). Of note, the high prevalence of warts related to HPV infection observed in patients with SCID-X1 after HSCT and gene therapy (128) has been suggested to be associated with persistent NK defects (52). However, the clinical consequences of NK cell deficiency has been a matter of debate (44, 129–131), and alternative hypotheses to explain the prevalence of these infections in SCID-X1 patients have been put forward, including defective γc signaling in keratinocytes (128). In conclusion, promising results have been reported with the use of LVs and conditioning treatment regarding the NK-cell reconstitution, although long-term follow-up and results from other clinical trials, hopefully including more phenotypic and functional studies, are needed to confirm them.

### Immune Reconstitution Differences After Gene Therapy *vs.* HSCT in SCID-X1 Patients

To date, only one published paper has directly compared immune recovery in SCID-X1 patients treated with gene therapy and HSCT (127), most likely due to the relatively low number of SCID-X1 patients treated with gene therapy so far. This was a retrospective study conducted in one center, which compared 13 patients treated with HLA-mismatched HSCT (receiving two doses of rabbit anti-thymoglobulin on days -2 and -1) and 14 patients treated with gene therapy (using 1^st^ generation γRVs or 2^nd^ generation SIN-γRVs in the absence of pre-conditioning, except one patient receiving fludarabine on days -3 and -2 to reduce the number of maternally engrafted T-cells). Overall, results showed faster T-cell reconstitution in patients treated with gene therapy *vs.* HSCT, with a high number of total T-cells, T CD4^+^ and T CD8^+^, as well as RTE cells (CD4^+^, CD45RA^+^, CD31^+^) at 6 and 12 months after treatment. However, the use of ATG prior to HSCT could also play a role in slower immune reconstitution in these patients. These differences continued being observed 5 years after the treatment for RTE cells, but disappeared for the other T-cell subsets (127). Rapid T-cell recovery during the first months after engraftment is essential to prevent accumulation of dangerous infections, which are one of the main causes of death in patients treated with HSCT (38).

On the other hand, no differences were observed in the distribution of B-cell subpopulations or IgA levels between both groups of patients, although IgM levels were higher 24 months after treatment in gene therapy group. Most likely due to the absence of conditioning, B-cell function remained defective in all patients, although in 4/12 patients treated with gene therapy it was possible to stop IgRT (all of them treated with 1^st^ generation γRVs) *vs.* in none of those treated with HSCT (127). Similarly, NK-cell reconstitution was not achieved in any of the patients evaluated from both groups, despite a higher number of cells was observed in those patients treated with gene therapy. The subset of CD56^dim^ CD16^+^ cells as well as the expression of NKp46 and NKG2D were reduced in both groups of patients. In contrast, CD56 expression was reduced in HSCT patients *vs.* healthy donors and patients treated with gene therapy (127).

In conclusion, this informative study demonstrated faster immune reconstitution in SCID-X1 patients treated with gene therapy *vs.* HSCT, especially of T-cells, leading to more rapid resolution of some infections and a shorter duration of infection-related hospitalization (127). One explanation for this superior immune reconstitution during the first month might be the absence of GvHD or the use of younger autologous stem cells (132). Nevertheless, larger multicenter studies including more patients, as well as different therapeutic approaches used in both HSCT and gene therapy should be carried out to confirm these results.

## Conclusions and Future Perspectives

Gene therapy treatments for patients with SCID-X1 have improved considerably in the last 20 years. The promising results of the first clinical trials (6, 58, 59) were overshadowed by the occurrence of T-ALL in several patients (7, 79). However, the development of new safer γRVs and application in new clinical trials have showed encouraging results, with similar efficacy *vs.* previous trials and absence of leukemogenesis so far (63). Most recently, the use of LVs and the introduction of low-intensity conditioning have also improved the safety and immune reconstitution (60), even in older patients (62). The introduction of conditioning has played a particularly important role in improved multilineage immune recovery in SCID-X1 and other PIDs, regardless of the vector used (60, 62, 103, 109, 113).

CD4^+^ and naïve CD4^+^ T-cell counts at 6 and 12 months after HSCT have now been identified as predictive biomarkers for the long-term T-cell reconstitution and overall survival in SCID patients (34). The discovery of these kind of markers in SCID-X1 patients after gene therapy might facilitate the identification of those cases in which the treatment is failing, and would allow rapid additional intervention through a new infusion of gene corrected cells or progression to conditioned allogeneic HSCT, preventing organ damage and other complications arising from insufficient immune reconstitution. On the other hand, T-cell reconstitution has been reported to be faster after gene therapy *vs.* HSCT in patients with SCID-X1 (127). If these results are confirmed with larger series, this might change the current place of gene therapy in treatment protocols for SCID-X1 (31). However, these studies are sometimes hampered by the lack of standardized guidelines for the evaluation of immune reconstitution. A possible solution could be the use of international recommendations, such as those recently published by the Pediatric Blood and Marrow Transplant Consortium for the screening and management of SCID patients after HSCT (124).

Huge efforts are being put into the development of superior viral vectors for clinical use. Thus, improvements in LV design and production, as well as the culture and transduction protocols have been developed (111, 133–135), which might help to achieve a more efficient and cheaper manufacturing (133, 134), as well as better immune reconstitution (135). Furthermore, the application of conditioning treatment based on biologic agents for stem cell depletion (e.g. monoclonal antibodies), with the objective to avoid the toxic effects of classical treatments, is very promising (55–57).

Finally, despite the encouraging results using vector-based gene therapy in SCID-X1 patients and the absence of leukemogenesis with the SIN-γRVs and SIN-LVs so far, concerns surrounding insertional mutagenesis as well as the goal to obtain physiological expression patterns, have driven the search of new alternatives. Accordingly, gene editing tools are being explored for the treatment of several monogenic diseases, including PIDs. Despite the absence of clinical trials using these approaches for SCID-X1 treatment so far, recent *in vitro* and *in vivo* pre-clinical studies using zinc-finger nucleases (ZFNs) (136, 137), transcription activator-like effector nucleases (TALENs) (138), RNA-guided nucleases (CRISPR/Cas) (139) and adeno-associated viruses (140) have shown very promising results. [Booth et al. (141), Zhang et al. (142), and Rai et al. (143) recently reviewed the state-of-the-art of gene editing tools in primary immunodeficiencies]. However, the reduced efficiency of homologous recombination in HSPCs is a potential hurdle to be overcome before successful clinical application can be contemplated, even where a powerful selective growth advantage exists for corrected lineages (136, 137, 142). Widespread application of *ex vivo* gene therapy treatments remains complex because of the need for HSPC manipulation using elaborate protocols and sophisticated facilities (144, 145). The use of *in vivo* gene therapy through the direct delivery of the viral vector has also been proposed as a possible alternative, with encouraging results obtained in SCID-X1 canine models (144–146).

In conclusion, gene therapy approaches for the treatment of SCID-X1 have advanced considerably since the first clinical trials were performed. Safer vectors have been developed and the introduction of conditioning treatments have produced very promising clinical outcomes with limited toxicities. Longer follow-up and study of a larger numbers of patients is necessary to fully evaluate the place of gene therapy alongside conventional HSCT. Furthermore, the reduction of costs and the worldwide accessibility are important practicalities to consider. Finally, the application of new tools such as gene editing have the potential to revolutionize the gene therapy field for the treatment of SCID-X1 and other devastating monogenic diseases.

## Author Contributions

EB and AT conceived the manuscript. All authors contributed to the article and approved the submitted version.

## Funding

EB is supported by a postdoctoral grant from Fundación Alfonso Martín Escudero (Spain). AT is supported by The Wellcome Trust (217112/Z/19/Z), and all authors are supported by National Institute for Health Research Biomedical Research Centre at Great Ormond Street Hospital for Children NHS Foundation Trust. Funds were received from University College London (UCL) for open access publication fees.

## Conflict of Interest

AT is a co-founder and consultant to Orchard Therapeutics.

The remaining authors declare that the research was conducted in the absence of any commercial or financial relationships that could be construed as a potential conflict of interest.
